# Supplementary material for: Complexome profiling of the Chlamydomonas psb28 mutant reveals TEF5 as an early PSII assembly factor
Source: Plant Cell. 2025 Mar 17;37(6):koaf055. doi: 10.1093/plcell/koaf055 (PMC12164590; doi:10.1093/plcell/koaf055)
Supplement: koaf055_Supplementary_Data [file koaf055_supplementary_data.zip › psb28_interactive.zip!/plots/intensity/qualityPlots/peptides/_Cre10.g452350.t1.1.html]

### Cre10.g452350.t1.1

Trivial name:   
SeqCoverage [median]: 62.10  
Mass: 16.34 kDa  
  
 

| Sequence | MissedCleavage | Mass [Da] | wtIntensity | muIntensity | Score |
| --- | --- | --- | --- | --- | --- |
| DGFQSNAPLPPSSSIEAANQEGAAGYVEDAVK | 0 | 3218.50 | 10461.13 | 24711.41 | 89.14 |
| EGFHVK | 0 | 715.37 | 0.00 | 6398.06 | 88.63 |
| GVVSGLVSEPSGTEPGAWTGAAGWK | 0 | 2399.18 | 4835.22 | 2269.50 | 76.42 |
| IFSPDDTK | 0 | 921.44 | 22275.85 | 51835.97 | 123.69 |
| IFSPDDTKVK | 1 | 1148.61 | 30685.56 | 51082.67 | 118.04 |
| ISHHEGPSRPFK | 1 | 1390.71 | 8771.95 | 14947.09 | 167.11 |
| NYPVNNFSGR | 0 | 1166.55 | 29351.88 | 80136.77 | 158.57 |
| VKNYPVNNFSGR | 1 | 1393.71 | 13732.83 | 22122.38 | 105.25 |
|  |  |  | 120114.4 | 253503.8 |  |
